# Case report: Germline BRCA 2 mutation in primary peritoneal carcinoma: a rare malignancy

**DOI:** 10.3389/fonc.2024.1428918

**Published:** 2024-09-03

**Authors:** Yan-Ying Huang, Yue Zhang, Wen-Jun Xie, Zhao-Dong Li

**Affiliations:** ^1^ Department of Pathology, Hangzhou Red Cross Hospital, Hangzhou, China; ^2^ Department of Radiology, Hangzhou Red-Cross Hospital, Hangzhou, China; ^3^ Department of Pathology, Zhejiang Hospital of Traditional Chinese Medicine, Hangzhou, China; ^4^ Department of Clinical laboratory, Hangzhou Red Cross Hospital, Hangzhou, China

**Keywords:** primary peritoneal carcinoma, CA125, epithelial ovarian tumor, BRCA 2, PARP

## Abstract

**Background:**

Primary peritoneal carcinoma (PPC) is a rare malignancy. Clinically, its histological morphology resembles that of epithelial ovarian tumors (EOC), often leading to misdiagnosis. Diagnosis and treatment of PPC are time-sensitive because of the rapidly progressive nature of the disease.

**Case report:**

Herein, we report the case of a 54-year-old woman who was initially diagnosed with ovarian cancer; however, extensive workup showed evidence of Müllerian PPC origin. Furthermore, the patient harbored a targetable BRCA mutation.

**Conclusion:**

The patient was treated with the BRCA-targeting agents and had a good prognosis after surgery.

## Introduction

Primary peritoneal carcinoma (PPC) is a rare malignancy of the peritoneum that was first described in 1959 by Swerdlow as a “mesothelioma of pelvic peritoneum” (1). All the abdominal and pelvic cavities exhibited diffuse cancerous changes (2, 3). Clinically, PPC in women resembles advanced epithelial ovarian cancer (EOC); histologically, the malignant cells seen in PPC are very similar to EOC, and the tumor markers cancer antigen-125 (CA125) is often elevated. This has resulted in diagnostic challenges. However, the ovaries of patients with PPC are rarely affected or are affected only on the surface (2, 4, 5). The three most common symptoms are abdominal distension (51.5%), abdominal pain (54.9%), and other gastrointestinal symptoms (18.6%). The symptoms of early-stage disease are unclear, and late-stage symptoms are similar to those of ovarian cancer, such as a large amount of ascites, an abdominal mass, intestinal obstruction, and significantly elevated serum CA125 levels. The prognosis of patients with PPC is poor. According to the National Comprehensive Cancer Network Clinical Practice Guidelines in Oncology, the therapeutic principle of PPC is similar to that of EOC, with surgery and chemotherapy being the main treatment regimens (6). The American Society of Clinical Oncology (ASCO) Guidelines (7, 8) recommends that patients diagnosed with PPC should undergo genetic testing for germline, somatic pathogenic, or likely pathogenic variants in BRCA1 (g/sBRCA1) or BRCA2 (g/sBRCA2), and patients with g/sBRCA1 or g/sBRCA2 genes should be treated with olaparib. PPC associated with g/sBRCA1 or g/sBRCA2 mutations show a high response rate to treatment with PARP inhibitors and have a better prognosis and longer PFS than those without BRCA mutations (8, 9). The benefit was greater in subgroups with tumor BRCA mutations and positive homologous recombination deficiency (HRD) status (7, 9). Therefore, patients should be referred for genetic risk evaluation, and germline and somatic testing. This recommendation for germline and somatic testing is intentionally broad, so that doctors have the latitude to order, regardless of the molecular tests they consider necessary, based on the evaluation of individual patients and their family history of cancer (6). Finally, germline and/or somatic BRCA1/2 testing can apprise the selection of maintenance therapies for patients.

## Case presentation

A 54-year-old woman presented at our gynecology department with a chief complaint of abdominal distension for 2 years, which had been aggravated by abdominal pain for 15 days. Two years previously, the patient visited a local hospital for treatment and underwent vaginal B-ultrasound, which indicated an unilocular-solid cyst of 2.8 cm in the left adnexal area. HPV testing was positive for HPV68, whereas colposcopy showed chronic cervicitis with erosions. The patient underwent symptomatic treatment. After 15 days, the abdominal distension worsened and the abdominal circumference gradually increased in size, accompanied by lower abdominal pain, which was obvious after defecation and affected her sleep and appetite. The patient was admitted to our clinic for further treatment. Abdominal B-ultrasonography performed at a local hospital revealed a large amount of fluid in the abdominal cavity (20 × 20 mm echoless area in the left annex area). Laboratory test results revealed that the CA125 levels were 887 U/mL and human epididymis secretory protein 4 (HE4) levels were 245.2 pmol/L, which were significantly elevated. No abnormal routine blood test results were obtained.

**Figure 1:**
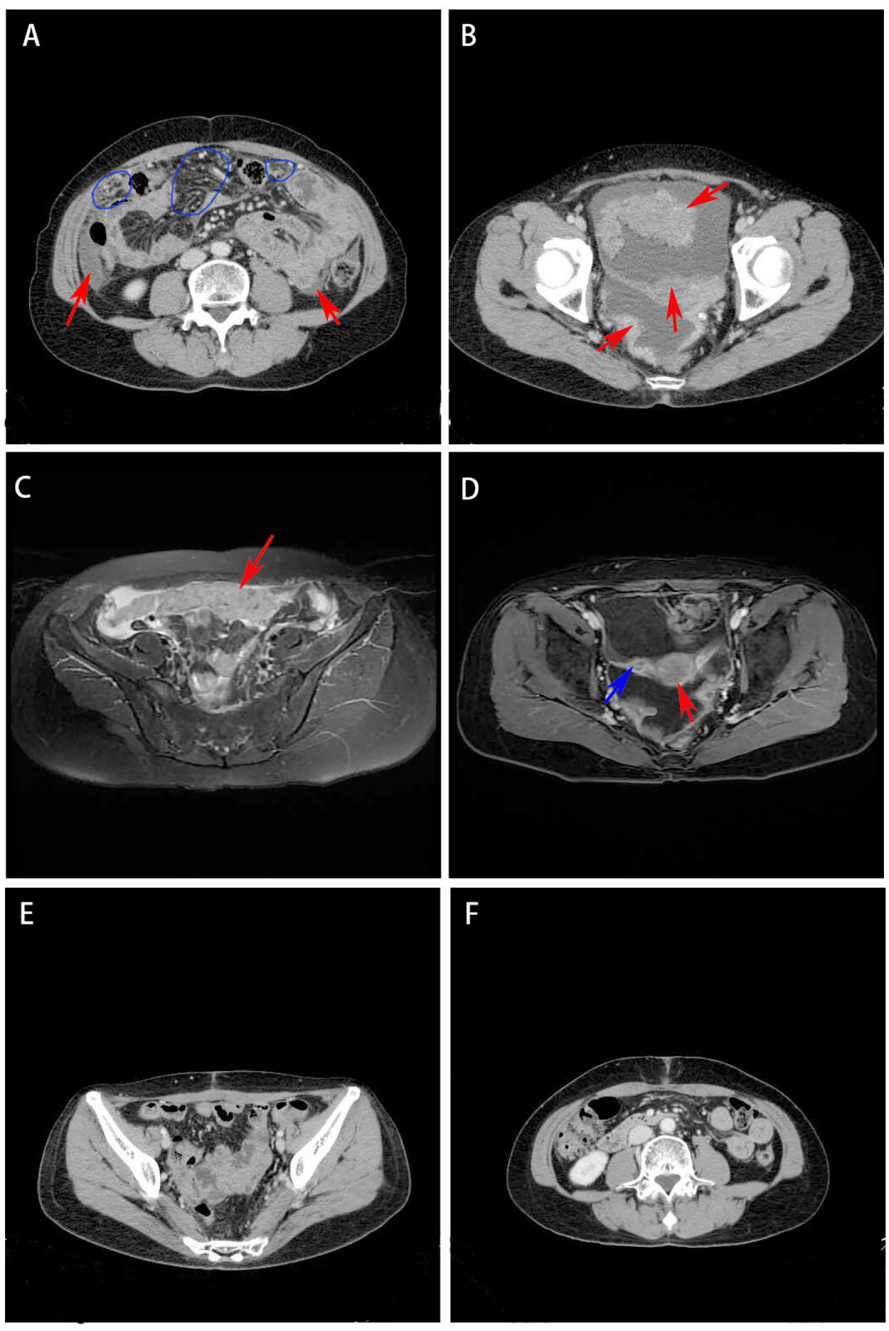
**(A)** CT revealed multiple small nodules (red arrow) and effusion (blue circle) in the abdominal cavity; **(B)** CT showed uneven pelvic floor peritoneum, nodular thickening, and obvious enhancement (red arrow); **(C)** Magnetic resonance imaging showed that the omentum changed in cake shape (red arrow); **(D)** Magnetic resonance imaging showed uterine (red arrow) and fallopian tube (blue arrow) involvement. **(E)** CT showed the peritoneal nodules disappeared and no obvious enhancement was observed; **(F)** CT showed that the small tubercles in the abdominal cavity were significantly reduced and the abdominal effusion had disappeared.

**Figure 2:**
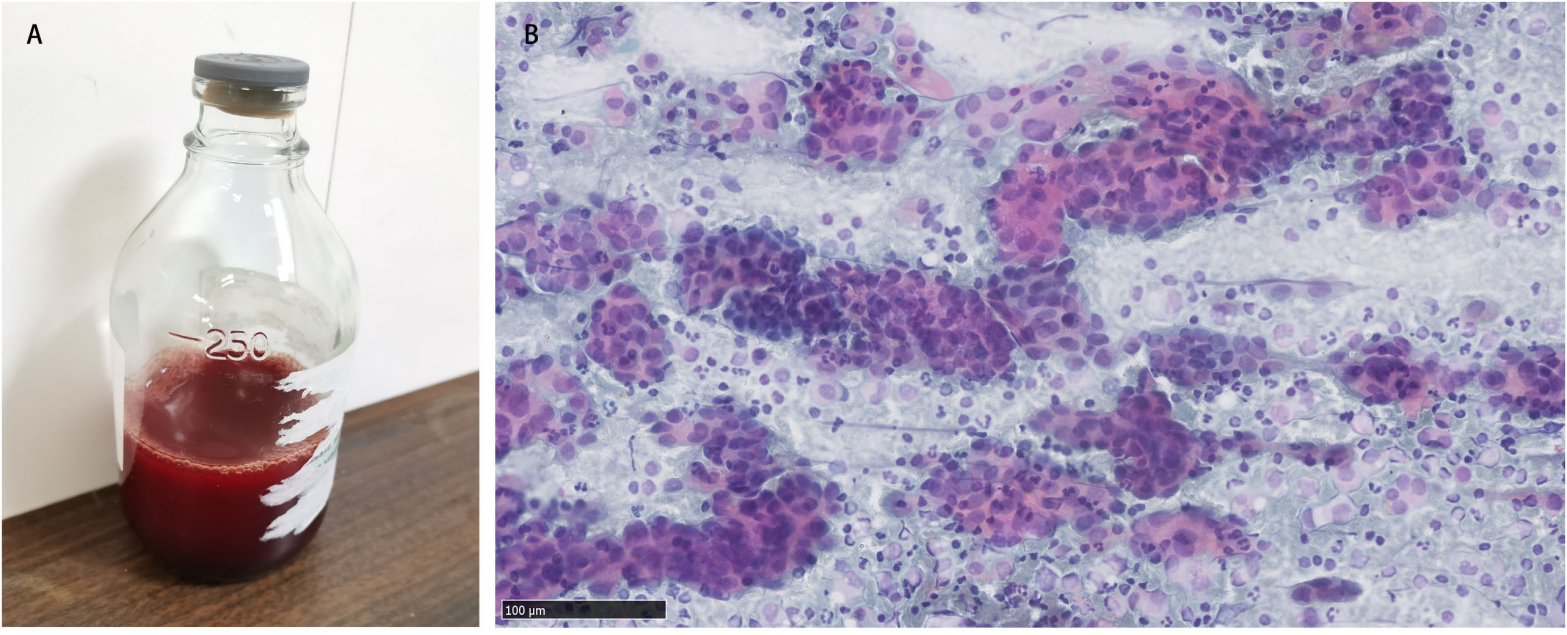
**(A)** Bloody ascites; **(B)** Cytological examination of ascites showed multiple clusters of adenocarcinoma cells.

**Figure 3:**
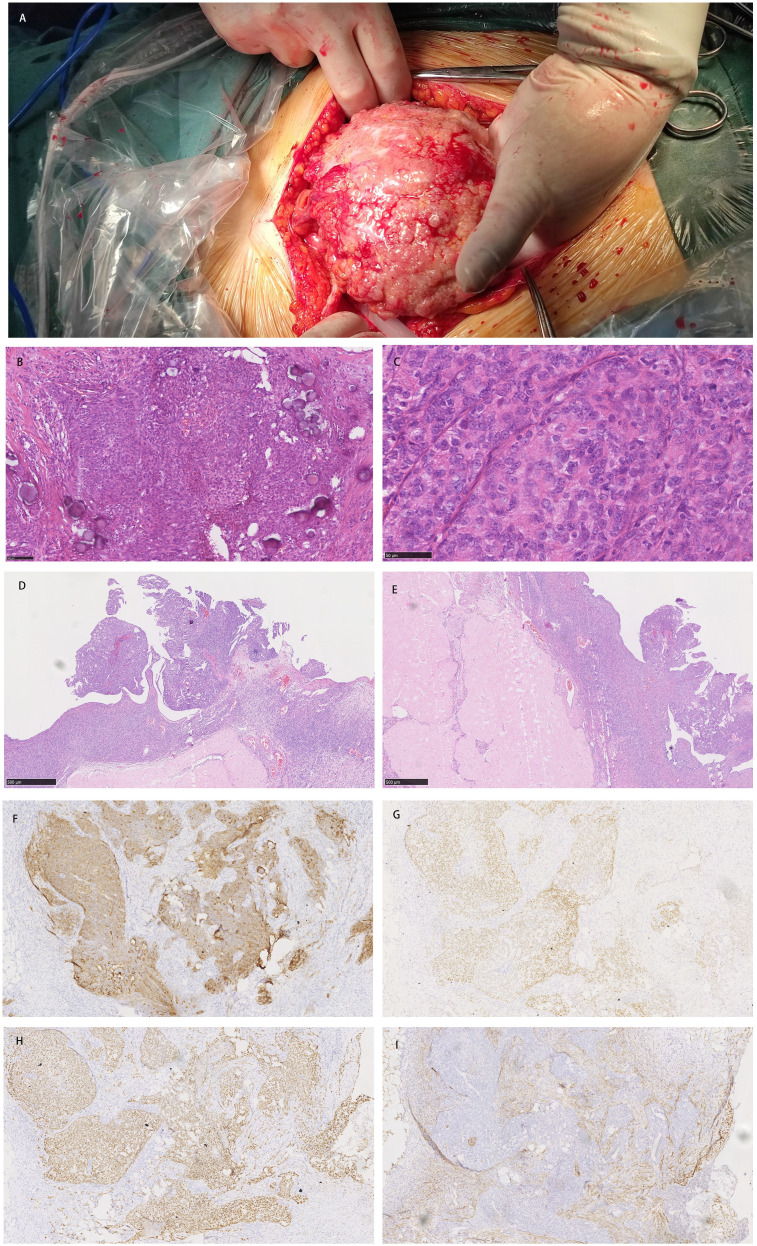
**(A)** Multiple nodules were observed in the greater omental tissue of the abdominal cavity. **(B)** Microscopy revealed epithelial tumors with necrosis and large amounts of sand-like calcification; **(C)** Tumor cell nuclei displayed marked pleomorphism and membrane irregularities; **(D)** One tumor involved the surface of the left ovary; **(E)** Another tumor involved the surface of the right ovary; **(F–H)** The expression of CA125, ER and Pax 8 markers was strongly positive; **(I)** The expression of calretinin was negative.

**Figure 4:**
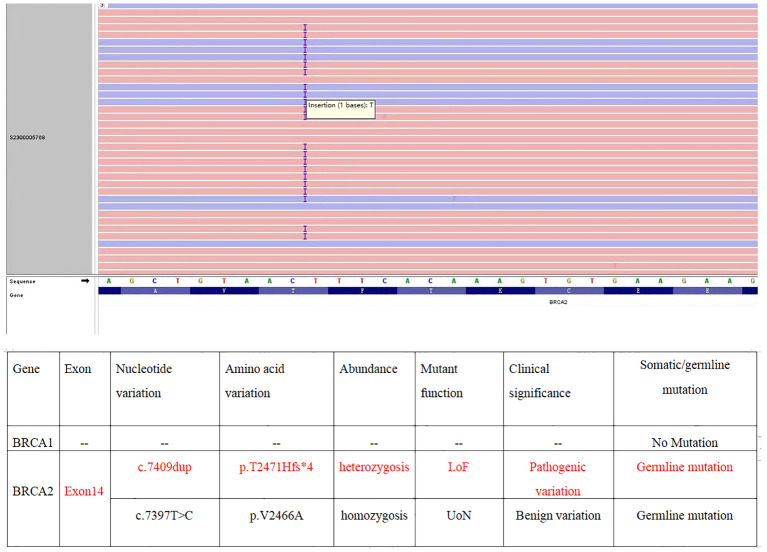
NGS showed BRCA2 mutation.

Gynecological examination at our hospital revealed a 6 × 5 cm hard nodule under the abdominal wall, and the uterus was palpated with ascites. Computed tomography (CT) revealed multiple small nodules and effusion in the abdominal cavity (**Figure 1A**), with an uneven pelvic floor peritoneum, nodular thickening, and obvious enhancement (**Figure 1B**). Magnetic resonance imaging showed an omental cake (**Figure 1C**) and uterine and fallopian tube involvement (**Figure 1D**). The CT revealed a mass in the left ovary, which prevented the complete exclusion of ovarian cancer. The ascites was punctured for cytological examination. The ascites was bloody (**Figure 2A**), and cytological examination of the ascites showed multiple clusters of adenocarcinoma cells (**Figure 2B**). According to the patient’s history, physical signs, and various examinations, malignant tumors were considered, and the indications for surgery were clear. Furthermore, there were no obvious contraindications for surgery. An emergency exploratory laparotomy was performed under general anesthesia. The following intraoperative findings were noted. There was approximately 3000 mL of effusion in the pelvis and peritoneal cavity, and the uterus and ovaries were atrophied on both sides, with several miliary lesions on the surface. The ovarian capsule was intact, and a 3 × 3 cm cyst was found on the mesofilm of the left fallopian tube. The appearance of the right fallopian tube did not differ. The omentum was adherent in a cake shape, approximately 20 × 15 cm in size, and hard in texture. Multiple nodules were observed in the greater omental tissue of the abdominal cavity (**Figure 3A**). The omentum was then removed using an ultrasonic knife. Large miliary lesions and small gray nodules were observed in both the left and right peritoneum and scattered nodular lesions were observed in the descending mesocolon of the transverse colon. After excision of the greater omentum, multiple hard lesions were found in the transverse mesocolon, ranging in size from 1 cm to 2 cm, and were resected using an ultrasonic knife. After probing the right lateral abdominal wall, the parietal peritoneum revealed a large miliary lesion tightly attached to the right ascending colon and wrapped in a mass. The appendix was not clearly displayed, and the tumor was sharply separated along its edge. The tumor was completely removed; its size was approximately 5 × 4 cm, and its texture was hard. After probing the left abdominal wall, large miliary lesions were found in the parietal peritoneum and scattered nodular lesions were found in the descending mesocolon, which were approximately from 0.5 cm to 1 cm in size. The lesions were resected using an ultrasonic knife, and the intestinal musculature was not involved. Intraoperative exploration revealed that the tumor lesions were widely planted in the patient, and miliary nodules could be palpated in the liver and spleen. However, because the liver and spleen were difficult to expose and resection could not be performed under direct view, laparoscopic exploration was performed during the operation, and electrocoagulation of the lesions under direct view was performed. Under laparoscopy, soybean-sized planting lesions and patchy miliary lesions could be seen in the retroperitoneum of the renal region at the location of the hepatorenal recess, Scattered miliary lesions can be found in the peritoneum above the left splenorenal recess, ranging in size from 4×2cm, and scattered miliary lesions can be found on part of the diaphragm. The lesions were resected using an ultrasonic knife. It was a CC-0 and the PCI score was 15. The entire procedure included total hysterectomy, double adnexectomy, pelvic lymph dissection, pelvic mass resection, partial mesenterectomy, greater omentectomy, appendectomy, peritoneal lesion resection, lesion electrocoagulation, and abdominal perfusion chemotherapy (cisplatin, 60 mg). After the abdominal cavity was rinsed with normal saline, each peel surface and stump were checked, and the bleeding was completely stopped. One drainage tube was placed in the basin, and two packets of gelatin sponge were placed locally to prevent bleeding. Thereafter, 60 mg + 100 mL of cisplatin was induced in the abdominal cavity, which was opened 3 h later. Gauze instruments were checked. The abdomen was closed by routine layering and a continuous intradermal suture with absorbable thread was performed. The surfaces of the transverse colon, peritoneal greater omentum, appendix, left and right pelvic peritoneum, right iliac fossa mass, surface mass of the colon, surface of the right ascending colon, and right ascending colon mass of the right abdominal wall were surgically removed. Microscopy revealed epithelial tumors with necrosis and large amounts of sand-like calcifications (**Figure 3B**). Tumor cell nuclei displayed marked pleomorphism and membrane irregularities (**Figure 3C**). One tumor involved the surface of the left ovary (**Figure 3D**). Another tumor involved the surface of the right ovary (**Figure 3E**). The expression of CA125, ER and Pax 8 markers was strongly positive (**Figures 3F–H**), whereas that of calretinin was negative (**Figure 3I**). Based on the clinical features and immunohistochemical results, our case met the diagnostic criteria for PPC IIIC of Müllerian origin. The patient underwent BRCA gene mutation detection and had a homologous recombination deficiency score of 25 for BRCA2 (reference value: 42) (**Figure 4**). Based on these results, the patient was considered sensitive to olapalil. Two courses of a TC regimen (270 mg paclitaxel and 600 mg carboplatin) were received on 03-06-2023 and 03-28-2023, with a smooth chemotherapy process. Subsequently, an additional TP regimen (paclitaxel 210 mg, d1 and cisplatin 50 mg, d1 and d8) was administered on 04-18-2023 and 05-10-2023 via intraperitoneal perfusion chemotherapy, and the patient’s condition stabilized after the treatment. On 06-14-2023, 300 mg of olapalil (BID) oral targeted maintenance therapy was administered. By this time, the serum CA125 level had dropped to 9.80 U/L. Follow-up CT examination showed that the peritoneal nodules had disappeared, and no obvious enhancement was observed on 2023-08-02 (**Figure 1E**). The CT examination also showed that the small tubercles in the abdominal cavity were significantly reduced, and the abdominal effusion had disappeared (**Figure 1F**). The patient began oral targeted maintenance therapy with olapalil 300 mg BID on 2023-06-14 and has been treated since then.

## Discussion

Decades after menopause, women show higher rates of PPC, which may be related to their long-term hormone levels. However, the incidence of PPC is low, whereas that of EOC is four times higher (10). Most patients with PPC are treated at an advanced stage and the therapeutic effect is not ideal. The survival of patients with PPC is shorter than that of patients with ovarian cancer, with a poor prognosis. Compared with patients with EOC, those with PPC tend to be older at diagnosis and occur almost exclusively in postmenopausal women. Patients with PPC usually present with nonspecific abdominal symptoms such as pain, bloating or fullness, nausea, vomiting, and a gradual increase in abdominal circumference. In terms of gross presentation, PPC are characterized by multiple soft tissue nodules along the peritoneal and greater omental surfaces, sometimes with larger or fused masses.

According to the histological classification of female genital tumors established by the World Health Organization, the pathological types of PPC include serous carcinoma, mucinous carcinoma, endometrioid carcinoma, clear cell carcinoma, transitional cell, mixed, and undifferentiated carcinoma (11). Serous carcinoma is the most common histopathological subtype of primary peritoneal serous papillary carcinoma, a rare PPC mainly found in the elderly and postmenopausal women. The Gynecologic Oncology Group has established the following diagnostic criteria for PPC (6): First, both ovaries are normal in size or enlarged by an unrelated condition. Second, extraovarian involvement must be greater than the involvement of the surface of either ovary, if any. Third, microscopically, the ovaries are not involved with the tumor or exhibit only serosal or cortical invasion with dimensions smaller than 5.0 × 5.0 mm. Fourth, the histopathological characteristics of the tumors should be predominantly serous. Based on these criteria, once a diagnosis of PPC is confirmed, surgical cytoreduction and chemotherapy are the main therapeutic options (6).

A previous study reported that 21.2% (11/52) of patients with PPC harbored BRCA mutations (12). Therefore, it is important to establish the BRCA1/2 mutation status of patients who may be eligible for maintenance therapy following the completion of platinum-based first-line chemotherapy. Olaparib maintenance therapy resulted in a higher progression-free survival (PFS) than placebo in patients with BRCA mutations. PARP inhibitors are particularly effective in patients with BRCA1/2 mutations and their use has been shown to prolong patient survival (13). Immunotherapy has shown promise in the treatment of advanced cancer; however, its role in the treatment of PPC remains unknown (14). Homologous recombination inhibitor maintenance therapy is advised for patients without BRCA1/2 mutations. In this case, the patient had a BRCA2 germline mutation, whereas her daughter tested negative for the serum BRAC gene mutation test, indicating that the mutant gene had not been passed on to her daughter. Our patient’s symptoms were greatly relieved and the prognosis was good with olaparib-targeted maintenance therapy.

## Conclusions

PPC is a relatively rare disease with a high rate of misdiagnosis. In particular, it is difficult to distinguish from advanced EOC. With improvements in surgical procedures, examinations, imaging technology, and pathology, our understanding of PPC is gradually expanding. However, owing to its low incidence, many clinical studies have encountered difficulties. In the future, multicenter cooperation may be necessary to further study the clinical characteristics and prognosis of PPC to achieve accurate diagnosis and treatment of this rare disease.

## Data Availability

The original contributions presented in the study are included in the article/supplementary material. Further inquiries can be directed to the corresponding author.
